# Natural Tannins as New Cross-Linking Materials for Soy-Based Adhesives

**DOI:** 10.3390/polym13040595

**Published:** 2021-02-16

**Authors:** Saman Ghahri, Xinyi Chen, Antonio Pizzi, Reza Hajihassani, Antonios N. Papadopoulos

**Affiliations:** 1 Wood and Forest Products Research Division, Research Institute of Forests and Rangelands, Agricultural Research, Education and Extension Organization (AREEO), Tehran 19395-1113, Iran; reza.hajihassani@gmail.com; 2 LERMAB, University of Lorraine, 88000 Epinal, France; xinyi.chen@univ-lorraine.fr (X.C.); antonio.pizzi@univ-lorraine.fr (A.P.); 3 Laboratory of Wood Chemistry and Technology, Department of Forestry and Natural Environment, International Hellenic University, GR-661 00 Drama, Greece

**Keywords:** tannin, soy protein, cross-linking material, wood adhesive, wood based composites

## Abstract

Human health problems and formaldehyde emission from wood-based composites are some of the major drawbacks of the traditional synthetic adhesives such as urea formaldehyde resins. There have been many attempts to decrease formaldehyde emission and replace urea formaldehyde resins with bio-based adhesives for wood-based composites. Because of some weakness in soy-based adhesive, chemicals have been used as modifiers. Modified soy-based adhesives without any formaldehyde have been successfully used to prepare wood panels. To achieve this, different synthetic cross-linking chemicals such as phenol formaldehyde resins and polyamidoamine-epichlorohydrin were used. However, in reality, what we need are totally green adhesives that use natural materials. In our previous research work, the use of tannins in combination with soy-based adhesives to make wood composites was investigated. Thus, in this research work, the feasibility of using three types of natural tannins (quebracho, mimosa and chestnut tannins) as cross-linking materials for soy adhesive was studied. The chemical bond formation and adhesion behaviors of tannin-modified soy adhesives were also investigated by Matrix-Assisted Laser Desorption/Ionization Time-of-Flight Mass Spectrometry (MALDI-ToF-MS) and thermo-mechanical analysis (TMA). The results showed that at ambient temperature, both ionic and covalent bonds formed between tannin constituents and amino acids; however, at higher temperature, covalent bonds are largely predominate. Based on the results obtained from the thermo-mechanical analysis, the modulus of elasticity (MOE) of soy adhesive is increased by adding tannins to its formulation. In addition, the chemical bond formation was proved by MALDI-ToF-MS.

## 1. Introduction

Soybeansare an ideal raw material for making wood adhesives because they are abundant, renewable, environmentally friendly and readily available [1]. Soy-based wood adhesives have many advantages such as low cost, easy handling and low pressing temperature [1]. Recently, researchers have done a considerable amount of work using different methods and adding different chemicals to soybean flour and soybean protein cross-linking as bio-based wood adhesive for wood-based composites [2,3,4,5,6].

Despite the great potential of soybeans—in the form of soy flour (SF) and isolated soy protein (ISP)—as non-formaldehyde adhesives for wood gluing, a major drawback is their low water resistance [7]. Some researchers have already focused themselves on improving the moisture resistance of these adhesives [8,9]. Several chemical modification strategies involving introduction of phenolic, thiol, maleyl, amine and hydroxyl groups into soy proteins have been attempted to improve the adhesive properties of ISP with limited success [10,11,12,13,14,15]. A number of cross-linking agents were also used to increase the water resistance of soy adhesives. To this purpose, several additives like polyamidoamine-epichlorohydrin (PAE), sodium dodecyl sulfate (SDS) and other synthetic materials like phenol formaldehyde (PF) resins have been used [16,17]. The main issue in using synthetic cross-linking materials for soy adhesive is their lower environmental friendliness and their dependence on fossil resources. Therefore, chemicals not depending on oil are needed to improve soybean flour while maintaining its acceptable adhesion properties. Consequently, there is great interest in substituting synthetic chemicals for soy adhesives modification to obtain high-performance bio-based adhesives.

Tannins are natural products obtained from plants and are very widespread in nature [18]. Tannins are, after lignins, a major source of polyphenolic components all over the world [19], but they have a considerably greater extraction potential than lignins. Vegetable tannins have been used to tan leather either alone or accompanied by other tanning agents for several thousand years. They are natural products obtained from plants and are very diffuse in the whole plant kingdom [18].

The term natural vegetable tannin is used loosely to define two broad classes of chemical compounds of mainly phenolic nature, namely, condensed or polyflavonoid tannins and hydrolysable tannins [18]. Industrial polyflavonoid tannin extracts are mostly composed of flavon-3-ols repeating units, and two types of phenolic rings having different reactivates with formaldehyde are present on each flavon-3-ol repeating unit, namely A-rings and B-rings, with each repeating unit being linked 4,6 or 4,8 with the units, which precede and follow it [20]. Previous research works showed that the addition of tannin to the materials based on the blends of collagen and chitosan leads to modification of several properties, such as water swelling and mechanical performance [21].

Both condensed polyflavonoid tannins and hydrolysable tannins have already been successfully used as wood composite adhesives [22,23,24,25,26,27,28,29,30,31].

Synthesized tannin resin from larch tannin extract with 60.1 wt% has already been successfully used as a modifier of ISP adhesive. The results obtained showed higher crosslinking density and better plywood water uptake resistance and shear strength as well [32]. Moreover, the results of cured ISP-condensed tannin bio-adhesive showed a high cross-linking density, which was attributed to two factors: first, that the imine group (H_2_C=N–CH_2_+) generated by hexamine decomposition reacted with the nucleophilic site of the tannin’s A-ring to form an amino methylene bridge [33]. The bridge had the ability to bond with other condensed tannins oligomers and finally increase the crosslinking density of the resultant adhesive. Secondly, the orthoquinone obtained via pyrocatechol oxidation on the tannin’s B-ring led to covalent interaction and cross-linking reaction with ISP molecules, which further increased the cross-linking density of the resultant adhesive [34].

Because of the tannin’s phenolic nature, due to their similarity to phenol in PF resins, tannins are a good choice for soy adhesive modification. In previous research, tannin-modified soy-based adhesives were successfully used for wood-based composites, and satisfactory results were achieved [35,36,37]. 

The problem of synthetic adhesives is outlined in the introduction. They are petroleum derived; hence, they are a finite source, and they use and emit formaldehyde, now classified as toxic and carcinogenic. Adhesives based on other aldehydes are slower reacting, thus the work outlined here is aimed at offering a totally bio-sourced environment adhesive for bonding wood panels.

The aim of this research work is to apply different net natural tannins (hydrolysable and condensed tannin) as cross-linking agents of soy adhesives (isolated soy protein and soy flour) and to show the great potential of tannins (quebracho, mimosa and chestnut tannins) as cross-linkers for soy adhesives which used for wood composite bonding.

## 2. Materials and Methods

Defatted soy flour (SF) with 47 wt% protein content was purchased from BEHPAK Co. (Iran), and isolated soy protein (ISP) with 90 wt% protein content used in this study was donated by ADM Co. (USA). The three tannins used were mimosa bark tannin (Acacia mearnsii, formerly mollissima, de Wildt) extract, chestnut wood tannin (Castanea sativa) and quebracho wood tannin (Schinopsis balansae) extract (produced by SILVA Chimica, S. Michele Mondovi, Italy). The other chemicals were supplied by ACROS Organics.

### 2.1. Adhesive Formulation

Adhesive formulations were described in previous research work [34]. Different amounts of SF and ISP (7.35 g) were added to distilled water (29.4 g) and mixed with a mechanical stirrer (700 rpm speed) at room temperature (21 ± 2 °C) to produce soy slurry with 20 wt% solid content. The tannin solution (5, 10 and 15 wt% based on soy dry weight) was then added to the soy slurry and stirred for 30 min. The tannin solutions in water were prepared at 45 wt% concentration. In addition, 6.5 wt% hexamethylenetetramine (hexamine) was used as a hardener; the percentage was based on the dry weight of tannins. Hexamine was added to the adhesives as a 40% aqueous solution. Finally, in the last stage of the adhesive’s preparation, the hardener was added and the pH adjusted to 7 with a 50 wt% NaOH water solution. ISP adhesive was prepared according to the same procedure.

### 2.2. Matrix-Assisted Laser Desorption/Ionization Time-of-Flight (MALDI-ToF) Mass Spectrometry

Mimosa tannin extract was reacted at ambient temperature and at 80 °C for 1 h with soy protein isolates (ISP). Test procedure was performed according to the method as previously described by Chen et al. [37]. Samples for MALDI-ToF analysis were prepared by first dissolving 7.5 mg of sample powder in 1mL of a 50:50 *v/v* acetone/water solution. Then 10 mg of this solution was added to 10 μL of a 2,5-dihydroxy benzoic acid (DHB) matrix. The locations dedicated to the samples on the analysis plaque were first covered with 2 μL of a NaCl solution 0.1 M in 2:1 *v/v* methanol/water, and pre-dried. Then 1.5 μL of the sample solution was placed on its dedicated location and the plaque was dried again. Red phosphorous was to standardize the MALDI equipment. MALDI-ToF spectra were obtained using an Axima-Performance mass spectrometer from Shimadzu Biotech (Kratos Analytical Shimadzu Europe Ltd., Manchester, UK) using a linear polarity-positive tuning mode [37].

### 2.3. FTIR Analysis

Fourier transform infrared (FTIR) analysis of the reacted product of tannin + ISP reacted at 80 °C for 1 h. The addition of an acid catalyst (pTSA) was carried out using a Shimadzu IR Affinity-1 (Shimadzu Europe Ltd., Manchester, UK) spectrophotometer. A blank sample tablet of potassium bromide, ACS reagent from ACROS Organics (Geel, Belgium), was prepared for the reference spectra. Similar tablets were prepared by mixing potassium bromide with 5% by weight of the sample powders. The spectra were plotted in percentage transmittance by combining 32 scans with a resolution of 2.0 cm^−1^ in the 400–4000 cm^−1^ range.

### 2.4. Thermomechanical Analysis

The adhesives were tested dynamically by thermo-mechanical analysis (TMA) on a Mettler 40 apparatus. Different samples of two beech wood plies, each 0.5 mm thick, bonded with each adhesive system, for sample dimensions of 17mm × 5mm × 1 mm, were tested in non-isothermal mode between 25 °C and 250 °C at a heating rate of 10 °C/min in three-point bending. A force varying continuously between 0.1 N, 0.5 N, and back to 0.1 N was applied on the specimens with each force cycle of 12 s (6 s/6 s). The classical mechanics relation between force and deflection E = [L3/(4bh3)][ΔF/(Δf)] (where L is the sample length, b and h are the sample width and thickness, ΔF is the variation of the force applied, and Δf is the deflection obtained) allows the calculation of the modulus of elasticity (MOE) E for each case tested. Different formulations of adhesives were tested by TMA [35].

## 3. Results and Discussion

**Figure 1:**
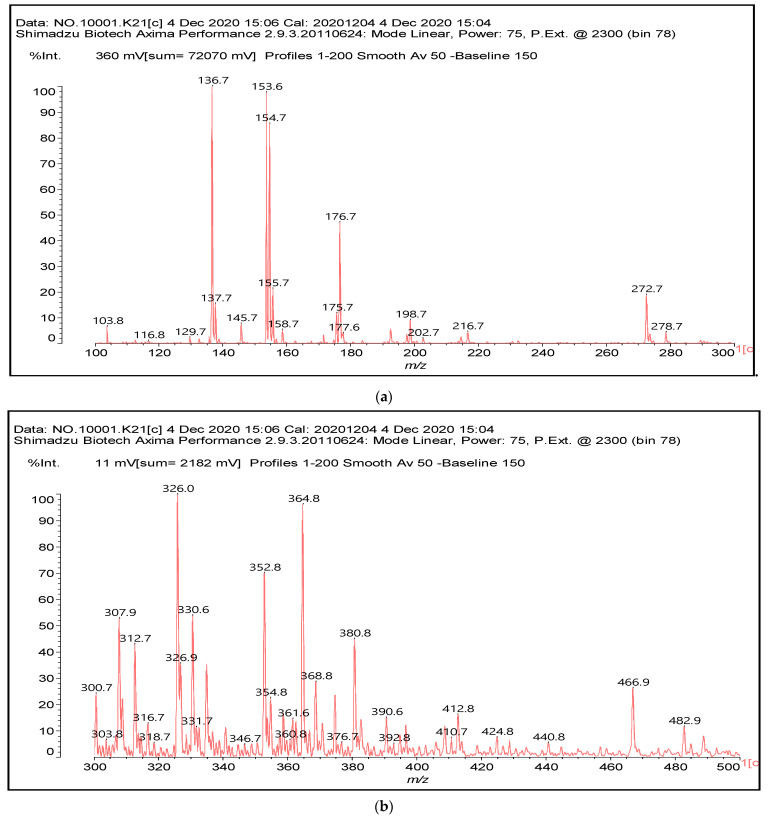

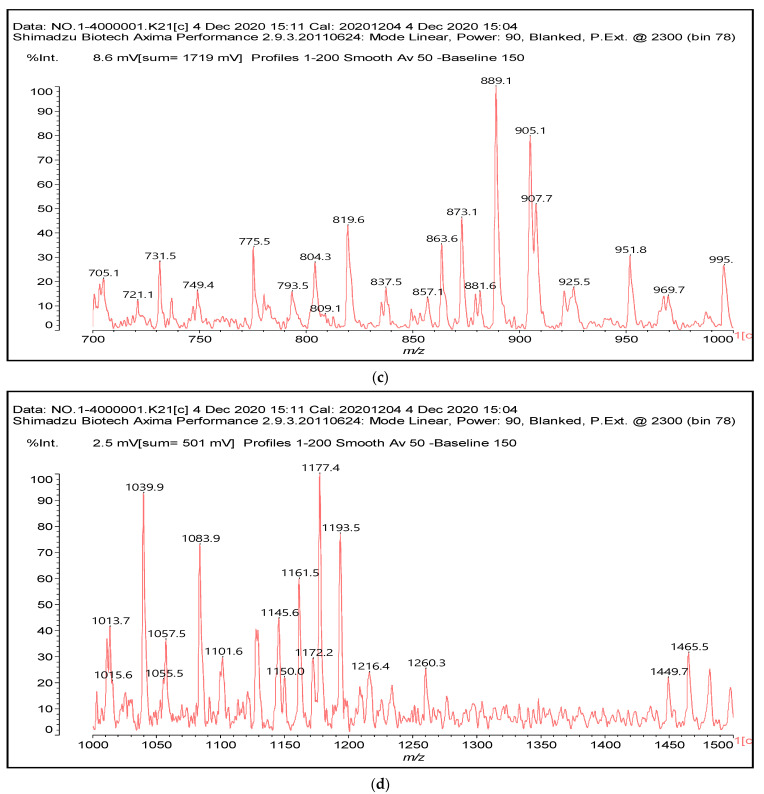
MALDI-ToF spectra of tannin-ISP adhesive. (**a**) 100–300 Da range. (**b**) 300–500 Da range. (**c**) 700–1000 Da range. (**d**) 1000–1500 Da range.

The main components found in the different tannin extracts used coincided with what was obtained by previous work by MALDI-ToF analysis [20,38,39]. Figure 1 shows the MALDI-ToF spectra for ISP modified by tannin extracts. The results indicate that new compounds have formed by reactions of different hydrolysable and condensed polyflavonoid tannin extracts with soy protein amino acids. The effect is particularly evident when the reaction is done at 80 °C with a condensed tannin where the predominant linkages are covalent while the ionic linkages present for reactions at ambient temperature have disappeared. The interaction between tannin constituents and amino acids occurs by reaction of the carboxyl group (-COOH) of gallic acid of chestnut tannin with amino groups of amino acids side chains. Quebracho and mimosa tannins react with amino acid functional groups and with the hydroxyl groups (-OH) on phenolic rings.

There are a number of compounds formed and detected by the MALDI-ToF analysis of tannin and ISP, as depicted in Figure 1. The MALDI-ToF spectra clearly show that amino acids are linked to flavonoid monomers and dimers. In addition, the results reveal soy protein polymer chains up to four amino acids linked to flavonoid monomers or dimers, and flavonoid dimers linked to two peptidic chains as well. Regarding covalent bonds, their formation is favored at high temperature. There are two main reactions occurring: one is the amination of the tannin, and this can happen with any amino group of an amino acid. The MALDI analysis indicates that many amino acids when alone react with tannin flavonoid monomers through their amino group. However, when the same amino group is linked as it is in the peptide chain of the protein, the only free amino groups that can react in this way are those of the side chain of arginine because they are not involved in the peptidic bond of the skeletal chain of the protein. The reactions of amines with tannins are well documented, it is facile, both using ammonia or primary amines and demonstrated with other techniques other than MALDI [40,41].

In the case of arginine, its side chain -NH2 groups can substitute the -OH of the flavonoids [40]. The reaction of the carboxylic acid groups of the side chains of aspartic and glutamic acid to form an ester is favored with the alcoholic -OH group on the C3 of the heterocyclic ring of the flavonoids. This is logical because, as the phenolic hydroxyl groups have more acidic character, they will be much less likely to react with the carboxylic acid groups of the side chains of aspartic and glutamic acids. Reaction with the phenolic hydroxyl groups, however, cannot be excluded, but the MALDI technique cannot ascertain it. It is also evident that this esterification reaction can really occur at the elevated temperatures experienced by the adhesive during adhesive hot pressing.

The MALDI-ToF analysis shows the peaks corresponding to the molecular weight of the amino acids and tannin extracts and molecular weight of new compounds resulting from bonds between them (with and without Na+) in Figure 1. In addition, the assignments for the peaks of the MALDI spectra are shown in Table 1. It must be stressed that the reaction products between single amino acids and a flavonoid monomer or dimer does not count for the cross-linking of the soy adhesive by the tannin because the reaction in these cases appears not to be able to occur on the –NH- or –COOH that are in the skeletal chain of the protein as these are blocked as they participate to the peptidic bond. Thus, the structures that do not count for cross-linking are of the type such as for the 390 Da peak, and many others (Table 1) that correspond to an open fisetinidin structure linked to leucine (without Na+)(I). This clearly shows covalent bond formation between a tannin extracts monoflavonoid(fisetinidin) with an amino acid -NH_2_ group substitution of the -OH of the flavonoids as follows:


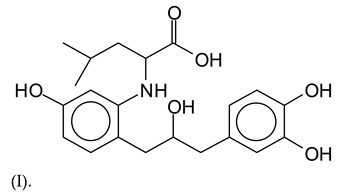


Equally, there are many peaks of single amino acids or peptide fragments that have not reacted with the flavonoids of the tannin. In addition, other peaks correspond to the several amino acids and tannin extract monomers and oligomers of different molecular weight such asthose at 131 Da, 146 Da, 154 Da, 156 Da, 175.7 Da, 202.7 Da, 204 Da, 273 Da, 326 Da. In addition, peptide two amino acids dimers such as arginine-leucine with Na+(II) were assigned in Table 1 from Figure 1a,b.


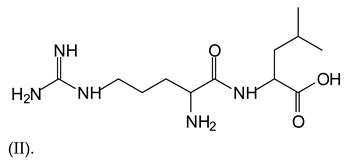


In the same type of peptide fragments the peak at 439 Da (III) assigned to the trimer of aspartic, leucine and arginine (with Na+) from the soy protein polymer chain (Table 1, Figure 1b) and others.


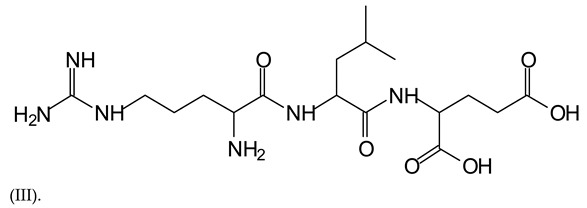


The type of linkages that will count for cross-linking the main body of the protein are, for example, the link represented by the peak at 467 Da (IV) assigned to a flavonoid robinetinidin linked to an arginine (with Na+) (Table 1, Figure 1b).


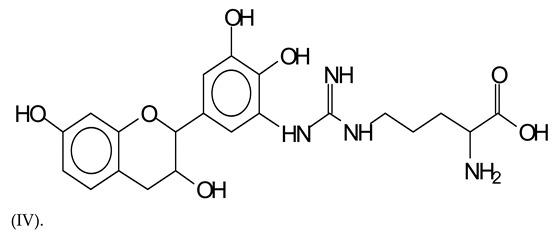


Similarly, the 483 Da (V) peak assigned to a delphinidin-arginine dimer (with Na+).


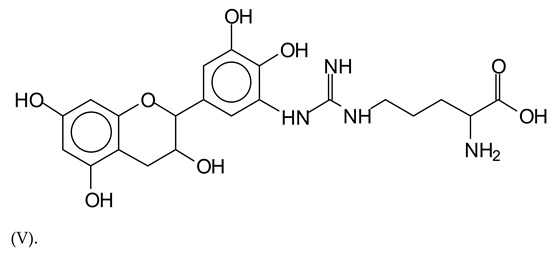


There are also several peaks in which a flavonoid is linked to a peptidic fragment containing arginine such as the peak at 575 Da, a leucine-delphinidin-arginine oligomer, no Na+ (VI).


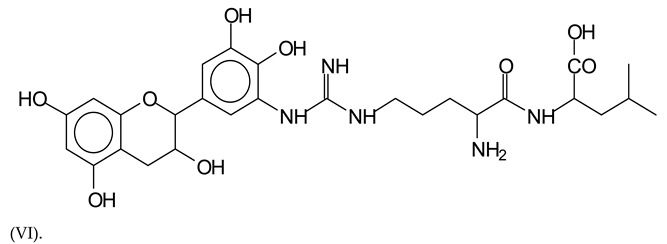


This shows that tannin flavonoid units are indeed linked and cross-link through the amino acids side chains of the main skeletal peptidic chain of the protein. There are many peaks and structures confirming this, such as the peaks at 601 Da, 632 Da, 688 Da, 705 Da, 857 Da, 873 Da, 889 Da, 1146 Da, 1161 Da, 1177 Da, 1194 Da, 1260 Da and 1449 Da (Table 1). For example, the species at 1449 Da (VII) indicates clearly that covalent cross-linking of the protein with a condensed tannin is indeed possible.


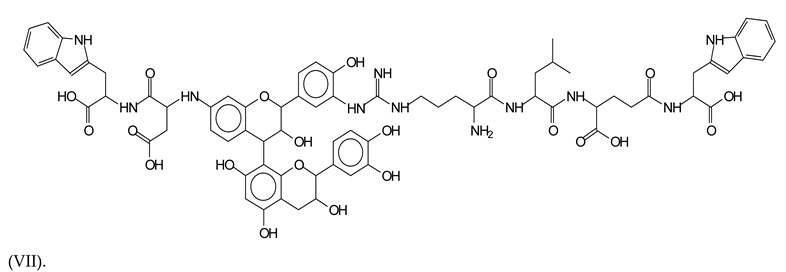


**Figure 2:**
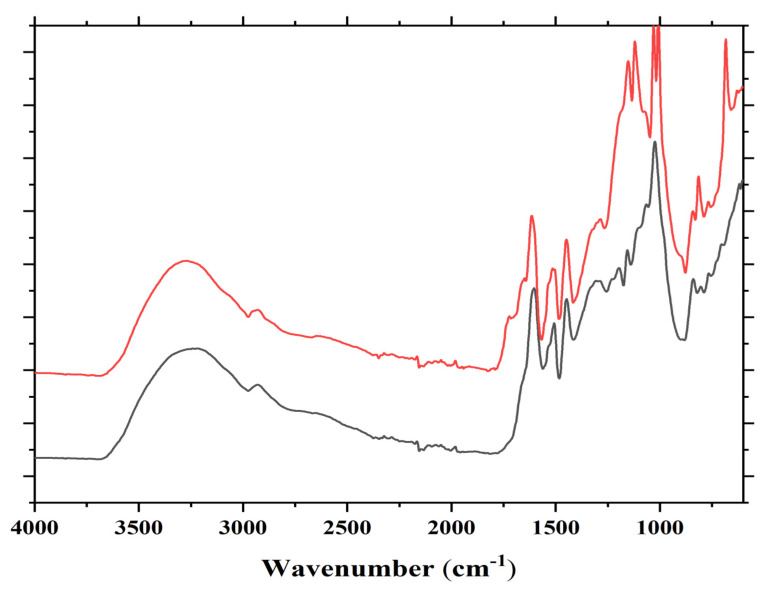
FTIR of reaction product mixture of condensed tannin with ISP at 80 °C without catalyst (lower spectrum) and with catalyst (higher spectrum) both at 80 °C.

Several bands are observed in the FTIR spectrum in Figure 2. Some are as follows: cm^−1^; 1735 cm^−1^ (shoulder, C=O esters), 1711 cm^−1^ (shoulder, C=O acid in C-C-COOH) 1680 cm^−1^ (C=O amide and C=O in -COOH of acid), 1625 cm^−1^ (primary amine asymmetric stretch R-CH2-NH2), 1530 cm^−1^(amide), 1480 cm^−1^(amide), 1333 cm^−1^ and 1292 cm^−1^ (secondary amine, R-NH-R), 1165 cm^−1^, 1150 cm^−1^, 1030 cm^−1^ (primary amine asymmetric stretch R-CH2-NH2), 1000 cm^−1^, 850 + 700 cm^−1^ (primary amine).

In the FTIR, there are indications that support what was found by MALDI ToF analysis, namely that reactions occur through the primary amines in ISP by forming secondary amines, this being indicated by the two characteristic bands at 1333 and 1292 cm^−1^. That esterification of the tannin hydroxyls by the protein side chain acids does also occur is demonstrated by the 1735 cm^−1^ shoulder. These bands appear to support the MALDI results that the reaction of the protein with the flavonoid units of the tannin can occur both through the amino group of a protein side chain or by esterification with the acid group of a side chain of the protein.

**Figure 3:**
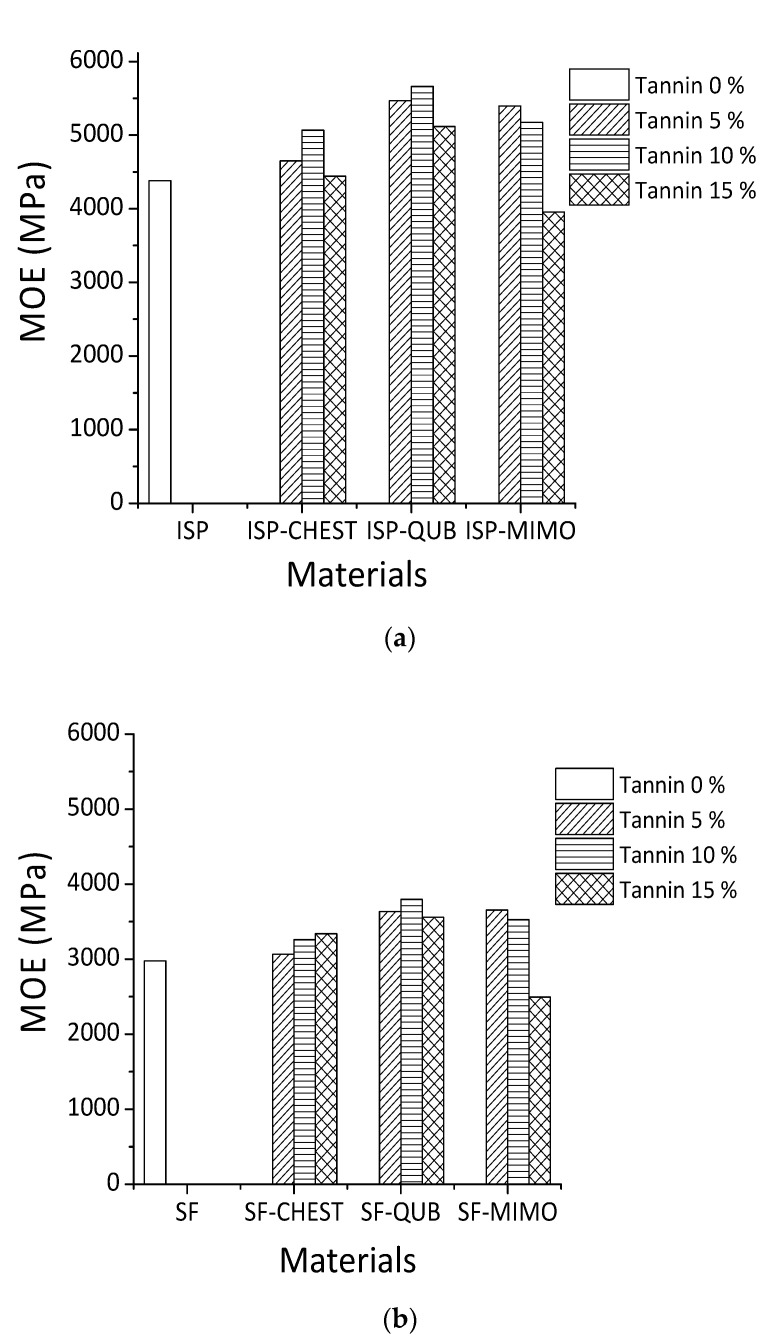
Effect of different tannins extracts on MOE of ISP (**a**) and SF (**b**).

The TMA results for different tannin-modified soy adhesives are shown in Figure 3. The influence of different constituents of chestnut tannin extract (Chest), quebracho (Qub) and mimosa (Mimo) tannins on the modulus of elasticity (MOE) of SF and ISP adhesives as a function of temperature is depicted in Figure 3a,b. When comparing the MOE content of different adhesive formulations, it is clear that the maximum MOE values of resins prepared with ISP are higher than those obtained with SF adhesive. The proportion of tannins added varied from 5% to 10% to 15% by weight. All the adhesives tested, show an increase in the maximum MOE value with increasing proportions of both hydrolysable and condensed. The highest MOE for both tannin-modified ISP and SF adhesives were obtained by the addition of 10% wt quebracho tannins (5660 MPa for ISP and 3799 MPa for SF), 5% wt mimosa tannin (5397 MPa for ISP and 3655 MPa for SF) and 10% wt to 15% wt chestnut tannin for ISP (5069 MPa) and SF (3341 MPa).

Based on the results obtained from MALDI-TOF mass spectrometry, covalent and ionic bonds can form between soy amino acids and tannins (Figure 3). For these reasons, the MOE of tannin-modified soy flour adhesive is increased. In previous studies on tannin and protein interactions, different bonds such as hydrogen, covalent, ionic and hydrophobic bonding between tannin and protein were reported [21,42,43], and the reaction between tannins and ammines has been recently studied and codified in depth [44]. Condensed polyflavonoid quebracho and mimosa tannins when compared to hydrolysable chestnut tannin showed better reaction with soy protein. This happened because of the higher reactivity of aromatic -OH groups in condensed tannins than the aromatic and aliphatic -OH groups in hydrolysable tannins. Between two industrial polyflavonoids, quebracho appears to react well with soy protein. Compared to the more branched structure of mimosa tannin oligomers, quebracho tannin oligomers are predominantly linear and the inter flavonoid link in quebracho tannin structure is more easily hydrolysable. These structural differences also contribute to the significant differences in reaction of two condensed tannins with soy (SF and ISP) in slurry systems.

## 4. Conclusions

Three types of natural tannins—chestnut tannin extracts, quebracho and mimosa condensed polyflavonoid tannins—were used as cross-linking agents for SF and ISP adhesives for wood bonding. MALDI-ToF mass spectrometry clearly shows that ionic and covalent bonding between amino acids and tannin constituents are formed at ambient temperature and that covalent bonds do predominate at elevated temperature as present in the hot-pressing of wood panels. They are the bonds exclusively between the –NH2 and –COOH groups of side chains of amino acids in the peptide chain that participate in the tannin/protein cross-linking; thus, the amino acids arginine, aspartic acid and glutamic acid are the only ones able to participate. The TMA results also indicate a higher MOE and better adhesion properties of soy adhesives by adding tannins to their formulation. The quebracho tannin showed higher MOE for both SF and ISP adhesives. Higher MOE was an evidence to confirm bond formation determined by MALDI-ToF. The results obtained from this research clearly show the potential of tannins as cross-linkers for soy-based adhesives to produce totally green adhesives for wood bonding.

## Figures and Tables

**Table 1:** Assignment of structures to relevant MALDI-ToF peaks from Figure 1a to Figure 1d.

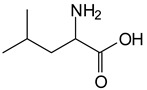 131 Da= Leucine, no Na^+^
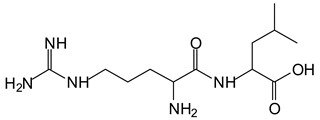 146 Da = glutamic acid, no Na^+^ 154 Da = Leucine with Na^+^ 155–156 Da = aspartic acid with Na^+^ 175.7 Da = no Na+, arginine, calculated 174.5 Da 202.7 Da = withNa+, Tyrosine, calculated 201.8 Da, OR no Na+,Tryptophan, calc. 204 Da 273 Da Fisetinidin, no NA^+^ 326 Da delphinidin +Na^+^ 308 Da = arginine-leucine with Na+ (calculate 310 Da)
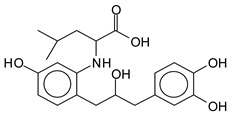 390 Da = Open fisetinidin-leucine no Na^+^(calculated 389.5 Da),
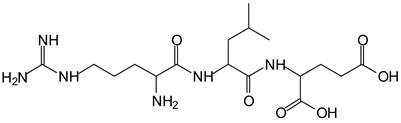 439 Da = aspartic-leucine arginine with Na^+^
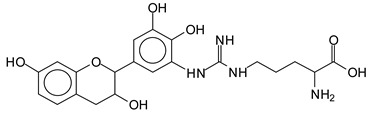 467 Da = robinetinidin-arginine with Na^+^,Calculated 469 Da
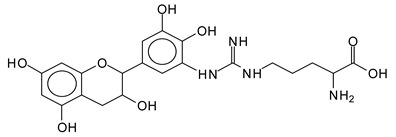 483 Da = delphinidin-argininewithNa^+^(calculated 485 Da)
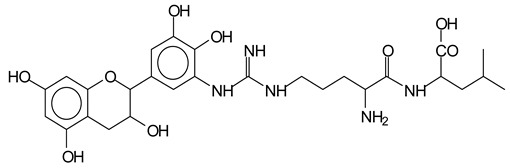 575 Da = leucine-delphinidin-arginine, no Na^+^, Calculated 575 Da
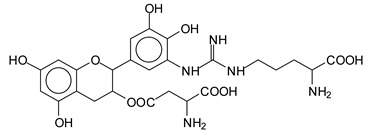 601 Da = aspartic acid-delphinidin-arginine, with Na^+^, calculated 601 Da.
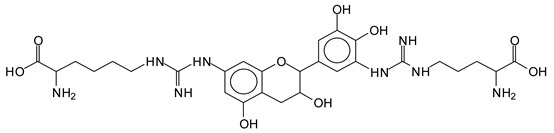 632 Da = arginine-delphinidin-arginine no Na^+^, calculated 632 Da
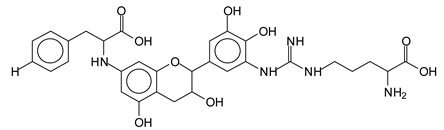 632 Da= Delphinidine-Arginine-Phenylalanine- with Na^+^, calculated
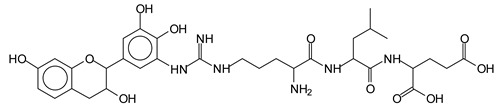 688.8 Da = no Na^+^, calculated 688.7 Da., robinetinidin-arginin-leucin-aspartic acid. Thus, the flavonoid attached to a peptide chain fragment.
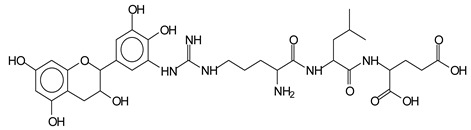 705 Da = no Na^+^, calculated 704.7 Da, delphinidin-arginine-leucin-aspartic acid. Thus, the flavonoid attached to a peptide chain fragment, with Na^+^, calculated 728.5.
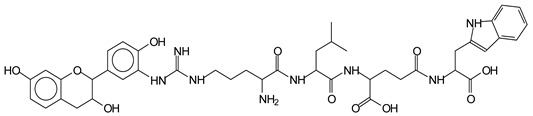 857 Da = no Na^+^, calculated 858 Da, fisetinidin-arginine-leucin-asparticacid-tryptophan. Thus, the flavonoid attached to a peptide chain fragment.
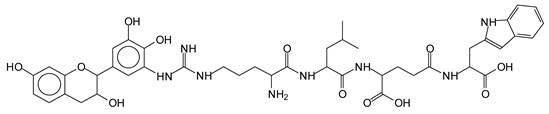 873 Da = no Na+, calculated 874 Da, robinetinidin-arginine-leucin-asparticacid-tryptophan. Thus, the flavonoid attached to a peptide chain fragment.
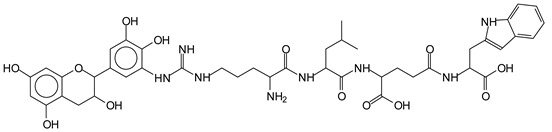 889 Da = no Na^+^, calculated 890 Da, delphinidin-arginine-leucin-asparticacid-tryptophan. Thus, the flavonoid attached to a peptide chain fragment.
1128 Da = no Na^+^, calculated 1129 Da, fisetinidindimer-arginine-leucin-aspartica cid-tryptophan. Thus, theflavonoid dimer attached to a peptide chain fragment.
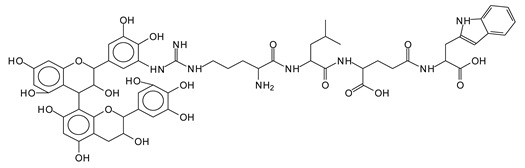 1194 Da =no Na^+^, calculated 1194 Da, delphinidindimer-arginine-leucin-asparticacid-tryptophan.Thus, the flavonoid dimer attached to a peptide chain fragment.
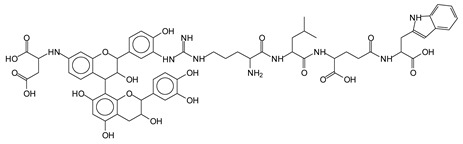 1260 Da = no Na^+^, calculated 1261 Da, aspartic acid-catechin-fisetinidin-arginine-leucin-aspartic acid-tryptophan. Thus, the flavonoid dimer attached to a peptide chain fragment + cross-linked with another aminoacid.
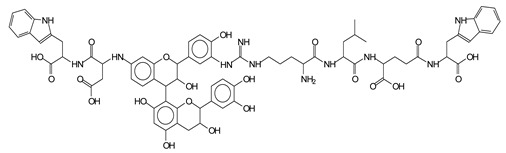 1449 Da = no Na^+^, calculated 1449 Da, Tryptophan-aspartic acid-catechin-fisetinidin-arginine-leucin-aspartic acid-tryptophan. Thus, the flavonoid dimer linked to two peptide chain fragments = cross-link protein tannin proved.

## Data Availability

The data presented in this study are available on request from the corresponding author.
